# Hyphal Index Following a Potassium Hydroxide Mount in Dermatophytosis

**DOI:** 10.7759/cureus.20576

**Published:** 2021-12-21

**Authors:** Abirami Chandramohan, Chakravarthi R Srinivas

**Affiliations:** 1 Dermatology, Kalinga Institute of Medical Sciences, Bhubaneswar, IND

**Keywords:** bacillary index, semi-quantification, dermatophytosis, hyphal index, potassium hydroxide mount

## Abstract

Dermatophytes are fungi that invade and proliferate over structures containing keratin. The potassium hydroxide (KOH) mount is a commonly performed investigation to detect the presence or absence of fungal elements. This study is an attempt to semi-quantify the KOH mount done for dermatophytosis and to validate the Hyphal Index (HI). HI is determined by the number of fungal elements present in the mount, in a method similar to the bacillary index in leprosy. This simple semi-quantification assessment is proposed as a prognostic tool in the management of dermatophytosis.

## Introduction

Superficial dermatophytosis is a rampant menace and is considered the current epidemic in the Indian sub-continent due to the humid climatic conditions and over-the-counter topical steroid abuse [1]. The potassium hydroxide (KOH) mount is a standard, quick, cost-effective investigation used to confirm the presence or absence of fungal elements in routine practice. The proposed Hyphal Index (HI) in KOH mount is akin to the bacillary index (BI) in slit skin smear (SSS) that is regularly carried out to supervise the trend of the patient with leprosy. The present binary style of KOH reporting does not monitor therapeutic progress as it does not record an increase or decrease in the fungal elements during treatment. This study is an attempt to semi-quantify KOH mount by grading the number of fungal elements and determining the HI. This article was previously presented as a poster at the 8th Mid Year Annual Conference of Indian Association of Dermatologists, Venereologists, and Leprologists (MID DERMACON 2020) on August 29-30, 2020.

## Technical report

An observational study of screening and diagnostic tests to validate the semi-quantification of fungal hyphae was conducted. The study was planned in the Dermatology outpatient department of a single tertiary care center located in Odisha, India. Considering a 26.7% prevalence rate of superficial dermatophytosis in India, the calculated sample size with 80% power at a 5% level of significance was 131 [2]. Ethical approval by the institutional ethical committee of the Kalinga Institute of Medical Sciences (approval no. 137/2019) was taken after which the study commenced from September 2019 to January 2021. Consenting patients with clinically diagnosed dermatophytosis were recruited into the study.

A 5×2 cm transparent cellophane tape was applied over a dermatophytic plaque, firmly pressed, and removed in a single swift motion. The tape was then stuck over a microscopic slide and the long end of the tape was gently lifted to add four drops of the 10% KOH solution [3]. After 30 minutes, the slide was initially examined under low power (40x) to scan the extent and distribution of the hyphae. The magnification was then gradually increased (100x and 400x) to confirm the presence of appropriate fungal load at particular fields. A magnification of 400x was used so that there was no misinterpretation of clumped and branching hyphae. The number of hyphae in each field was counted and the Hyphal Index was reported (Table 1).

**Table 1:** Semi-quantification of fungal hyphae in the potassium hydroxide (KOH) mount using Hyphal Index

KOH mount finding (per 100 fields)	Grading according to the Hyphal Index
Absence of fungal hyphae	0
Presence of up to 4 hyphae	1+
Number of hyphae present between Hyphal Index 1+ and 3+	2+
Presence of multiple hyphae	3+

The size of the field was the area visualized at 400x. This is different from the assessment of BI wherein the readings are taken in 1000x magnification under oil immersion. However, 1000x magnification was non-essential for the identification and quantification of the fungal elements considering the ample visualization at 400x due to the relatively larger dimensions of the fungus as compared to bacilli.

On the scanner mode, if there were significant fungal elements, up to five fields were examined to confirm the high HI, which is akin to BI wherein the identification of globi is given a BI 6+. However, when the fungal elements appeared scarce on the scanning mode, a diligent examination of 100 fields was done so that appropriate HI grading was awarded, which is similar to the BI [4].

Considering the increased number of patients presenting with tinea corporis, 180 consenting participants were recruited during the study period. KOH mount findings as found in them were analysed.

## Discussion

Out of the 180 mounts, 18 (10%) had no identifiable fungal elements in them and were thereby graded as HI 0. Sixty mounts (33.33%) showed up to four hyphae on examination of 100 fields and were marked with HI 1+. KOH mounting of 78 patients (43.33%) was graded as HI 2+ as the presence of fungal hyphae was between HI 1+ and 3+. Grade HI 3+ was found in 24 (13.33%) mounts wherein the fungal elements were numerous and uncountable. This data cannot be compared as, to our knowledge, no previous studies have been done with the Hyphal Index.

However, the proposed grading has been used in patients who underwent antifungal therapy wherein we observed a decrease in the HI on follow-up that suggests a possible correlation between the HI and the status of the disease (Original study: Abirami C, Srinivas CR. A Comparative Study of Hyphal Index in KOH Mount Pre and Post-treatment in Superficial Dermatophytosis).

The time taken to scan through 100 fields may be cumbersome posing as a limitation. Hence, staining of the KOH mount may facilitate swifter and precise readings.

## Conclusions

BI is a diagnostic and prognostic test wherein *Mycobacterium leprae* bacteria are microscopically counted, and is used to monitor response to treatment in Hansen's disease. Similarly, we recommend the use of HI to monitor the course of dermatophytosis and its response to treatment. This will aid in the prompt identification of ineffectiveness of a particular treatment, reducing the cost and time taken to treat the disease. Thus, semi-quantification may help in monitoring the effectiveness and also in comparing the relative effectiveness of two different drugs in the management of dermatophytosis.
